# Balloon dilation for failed pyeloplasty in children?

**DOI:** 10.1590/S1677-5538.IBJU.2018.0407

**Published:** 2019-07-27

**Authors:** Haifeng Duan, Wei Zhu, Wen Zhong, Xiaohang Li, Guohua Zeng

**Affiliations:** 1 Department of Urology, Minimally Invasive Surgery Center, The First Affiliated Hospital of Guangzhou Medical University, Guangzhou, Guangdong, China; 2 Guangdong Key Laboratory of Urology, Guangzhou, Guangdong, China

**Keywords:** Dilatation, Kidney, Child

## Abstract

**Objective:**

Pyeloplasty is considered the gold standard treatment for ureteropelvic junction obstruction (UPJO). However, the failure rate of pyeloplasty is as high as 10% and repeat pyeloplasty is more difficult. This study aimed to evaluate the efficacy of balloon dilatation for failed pyeloplasty in children.

**Materials and Methods:**

Between 2011 and 2017, 15 patients, aged 6 months to 14 years, were treated with balloon dilation for restenosis of UPJO after a failed pyeloplasty. Ultrasound and intravenous urography were used to evaluate the primary outcome. Success was defined as the relief of symptoms and improvement of hydronephrosis, which was identified by ultrasound at the last follow-up.

**Results:**

All patients successfully completed the operation, 13 patients by retrograde approach and 2 patients by antegrade approach. Thirteen patients were followed for a median of 15 (4 to 57) months and 2 patients were lost to follow-up. Resolution of the hydronephrosis was observed in 5 cases. The anteroposterior diameter (APD) of the pelvis decreased by an average of 12.4 ± 14.4mm. Eight patients needed another surgery. The average postoperative hospital stay was 1.78 ± 1.4 days. Two patients experienced fever after balloon dilation. No other complications were found.

**Conclusions:**

Balloon dilatation surgery is safe for children, but it is not recommended for failed pyeloplasty in that group of patients, owing to the low success rate.

## INTRODUCTION

UPJO is the most common pathological cause of neonatal hydronephrosis (1), with an overall incidence of 1: 1500. UPJO may lead to lumbago, urinary infection and renal dysfunction. The European Association of Urology Guidelines recommend pyeloplasty as the gold standard treatment for UPJO. Nevertheless, the failure rate of pyeloplasty may exceed 10% (2). Dy (3) reported a large survey in which approximately 11.4% of children required reoperations after pyeloplasty. Balloon dilatation is a minimally invasive treatment which is associated with low complications rate, and affords early recovery. Balloon dilations has a success rate of approximately 25-83% for the treatment of primary UPJO (2, 4, 5). Nevertheless, studies of balloon dilatation for failed pyeloplasty in children have been rare. Therefore, we conducted this study to assess whether balloon dilation is effective in treating failed pyeloplasty in children.

## MATERIALS AND METHODS

Between 2011 and 2017, 15 patients (14 boys, 1 girl), with a mean age of 3.4 years (range 0.5-14 years), were treated with balloon dilation for restenosis after pyeloplasty. Twelve cases were on the left side and 3 were on the right. Ten cases were open pyeloplasties, 4 were laparoscopic and 1 was robot-assisted.

The surgical indications were as follows: 1 - two ultrasounds (with a 1-week interval) showing increased hydronephrosis; 2 - intravenous urography revealing that the contrast agent was blocked at the UPJ. An examination of urine culture, urine routine and blood tests were performed before the operation. If patients had a positive urine culture, they would receive sensitive antibiotic therapy for one week, depending on the urine culture results.

Technique: patients were placed in the lithotomy position and all surgeries were done under general anesthesia. First, a 5-Fr ureteral catheter was inserted under the guidance of an ureteroscope to perform retrograde pyelography. Second, a 0.014” guidewire was inserted through the UPJ retrogradely or anterogradely (if retrograde insertion failed). A 6-Fr balloon catheter (X--Force U30, Bard, USA) with a 4cm balloon was introduced, and the balloon was placed across the stenotic segment with a guidewire, under X-ray guidance. The UPJ was fully expanded by injecting a radiopaque contrast medium when the pressure rose to 25-30 atm, and the pressure was maintained for 3-5 minutes. Finally, a 7-Fr double J stent was inserted into the ureter and was withdrawn by cystoscopy after 3 months. Broad-spectrum antibiotics were used to prevent urinary tract infection before and after the surgery.

The efficacy evaluation was as follows: ultrasound and intravenous urography were performed for all patients. Success was defined as the improvement of hydronephrosis after the double J (DJ) stent was withdrawn and a lack of recurrence during follow-up. Failure was defined as the need for another surgical intervention, including stent placement, endopyelotomy or pyeloplasty.

## RESULTS

Fifteen patients were treated with balloon dilation for the restenosis of UPJO. The mean age was 3.4 ± 3.7 years. The median time of the recurrence was 5 (From 1 to 35) months. Two patients were lost to follow-up. Thirteen patients were followed for an average of 21 months. Seven patients had low back pain and 2 patients had fever before surgery. Eight (61.5%) patients had positive preoperative urine cultures among which gram-negative bacteria predominated.

Thirteen children underwent retrograde balloon dilation, and 2 underwent the procedure via an anterograde percutaneous approach because of failure of retrograde guidewire insertion. Two patients experienced postoperative fever (> 38.5 degrees centigrade). The average postoperative hospital stay was 1.78 ± 1.4 days. Four patients had low back pain relief.

The improvement of renal hydronephrosis was observed in 5 cases (38.5%, after withdrawal of DJ stent, at 38, 30, 13, 5 and 4 months). The anteroposterior diameter (APD) of the pelvis decreased by an average of 12.4 ± 14.4mm.

Failures were observed in 8 cases. Among the 8 failure cases, 1 patient underwent another laparoscopic pyeloplasty and had a good result. Three patients chose another balloon dilation, and 3 patients underwent placement of an indwelling DJ stent. Currently, these 6 patients still have their DJ stents, because the APD increased substantially after withdrawal of the DJ stent. Nephrectomy was performed in 1 case in a different hospital.

## DISCUSSION

UPJ is defined as impaired urine flow from the pelvis into the proximal ureter with subsequent dilatation of the collecting system and potential damage to the kidney. The causes of UPJO can be classified into three types: 1 - stenosis in the lumen (the most common cause); 2 - dynamic obstruction; and 3 - outer lumen compression (common in ectopic vascular riding).

There are two types of treatments for UPJO: 1 - pyeloplasty (open, laparoscopic, robotic- -assisted); and 2 - endourological (anterogradely or retrogradely balloon dilatation, indwelling DJ tube, cold-knife, electrocautery or laser incision). Open pyeloplasty is the gold standard treatment of UPJO because of its high success rate of 90-100%. Laparoscopic and robotic-assisted pyeloplasty are becoming more popular, with advantages of minimal invasiveness and similar success rates (2, 6).

Robotic-assisted pyeloplasty has an advantage of shorter operation time and hospital stays (7, 8). Lucas et al. collected 865 cases and concluded that previous endopyelotomy and intraoperative crossing vessels increased the need for secondary procedures (9). Niver et al. compared the outcomes of robotic-assisted surgery for primary UPJO and secondary UPJO. They included 117 patients and concluded that robotic-assisted laparoscopic pyeloplasty was a safe and effective option for secondary UPJO repair (10).

Endourological surgery (including retrograde balloon dilatation, indwelling DJ tube, cold-knife and laser incision) can be performed through the urinary tract without any incision and therefore with faster recovery (11, 12), an attractive option for urologists. The reported success rates of endourological surgeries are inconsistent. Lewis-Russell reported a success rate of 83% in 40 patients based on renogram, after balloon dilation (13). However, Lin et al. (14). reported a success rate of only 30% in 9 patients. Cohen (15) reported a success rate (defined as resolution of obstruction radiographically or disappearance of symptoms) of 73% in 15 patients. Osther (16) showed that the balloon dilation success rate was 57% in 29 patients with congenital UPJO, but the success rate was only 25% in children. Factors such as presence of crossing vessels, stricture greater than 1.5cm and poor renal function are considered to be undesirable (17). For the treatment of patients with restenosis after pyeloplasty, Braga (18) reported that 18 children were treated with endourological treatment (including holmium laser and balloon dilation) and the success rate was 39%, similar to the value in the present study.

We chose ultrasound as the main follow-up and efficacy judgment tool for the dynamic evaluation of changes in hydronephrosis, as well as for postoperative follow-up, because ultrasound lacks radiation, and it is convenient and affordable; therefore, ultrasound is easily approved by parents. Park et al. followed up 215 patients at least 5 years by B ultrasound and reported that once hydronephrosis showed improvement, no recurrences were observed (19). Nevertheless, their results could have been affected by the subjectivity of the sonographer. Kurtz (20) also found that urinary tract infections could lead to the increased hydronephrosis. If urinary B ultrasound shows increased hydronephrosis, we suggest repeating the B ultrasound, not performing surgery immediately.

Song et al. (21) found that the incidence of urinary tract infections (UTI) in children with primary ureteral strictures was 36.2%. Children with failed pyeloplasties may have a higher urinary tract infection rate because of the series of iatrogenic operations. In our study, the rate of positive urine culture was 61.5% (8 of 13). Therefore, we should pay more attention to diagnosis and treatment of UTI.

There are several potential limitations to our study. First, we only assessed renal function by renal dynamic imaging in several patients who had severe hydronephrosis before surgery. Due to the cost and radiation, the examination of renal dynamic imaging was not accepted by most parents. Second, the length of the strictured segment that was considered an adverse factor for prognosis was not recorded. Finally, the number of cases was insufficient.

## CONCLUSIONS

Balloon dilation has the advantages of fast recovery, few complications and lack of incision; nevertheless, it is not recommended for children with failed pyeloplasties because of the low success rate.

## ABBREVIATIONS

UPJO: Ureteropelvic Junction Obstruction
APD: anteroposterior diameter of the pelvis
DJ stent: double J stent
Atm: atmosphere
UTI: urinary tract infection
